# Evaluation of the Cariogenic and Anti-Cariogenic Potential of Human Colostrum and Colostrum-Derived Probiotics: Impact on *S. mutans* Growth, Biofilm Formation, and *L. rhamnosus* Growth

**DOI:** 10.3390/life13091869

**Published:** 2023-09-05

**Authors:** Samaa A. Zaghloul, Sara N. Hashem, Safaa R. El-Sayed, Mona Shaban E. M. Badawy, Sarah I. Bukhari, Heba Mohammed Refat M. Selim, Omnia Karem M. Riad

**Affiliations:** 1 Department of Operative Dentistry, Faculty of Dental Medicine for Girls, Al-Azhar University, Cairo 11765, Egypt; samaazaghloul.26@azhar.edu.eg; 2 Department of Pedodontics and Oral Health, Faculty of Dental Medicine for Girls, Al-Azhar University, Cairo 11651, Egypt; safatolba.26@azhar.edu.eg; 3 Department of Microbiology and Immunology, Faculty of Pharmacy for Girls, Al-Azhar University, Cairo 11884, Egypt; monabadawe1657.el@azhar.edu.eg (M.S.E.M.B.); dr_omniakarem@azhar.edu.eg (O.K.M.R.); 4 Department of Pharmaceutics, College of Pharmacy, King Saud University, Riyadh 11451, Saudi Arabia; sbukhari@ksu.edu.sa; 5 Department of Pharmaceutical Sciences, Faculty of Pharmacy, Al-Maarefa University, Diriyah, Riyadh 13713, Saudi Arabia

**Keywords:** biofilm, breast feeding, cell-free supernatant, dental caries, human colostrum, *L. rhamnosus*, oral microbiota, probiotics, *S. mutans*, teeth decay

## Abstract

Human colostrum (HC) is essential for oral health as it is rich in probiotics that could affect the growth of the cariogenic *S. mutans* and its biofilm formation; hindering dental caries in advance. In this study, HC was collected from 36 healthy mothers 1–3 days postpartum. The effect of HC on oral health was carried out by assessing the impact of HC and its derived probiotics’ cell-free supernatants (CFS) on the growth of *S. mutans* (using modified well diffusion) and its biofilm formation (using microtiter plate assay). Moreover, the effect of whole HC on *L. rhamnosus*, a probiotic oral bacterium, was examined. Probiotics were isolated and identified phenotypically by API 50 CH carbohydrate fermentation and genotypically by 16S rRNA amplification. The in vitro study revealed that HC has cariogenic activity and is associated with biofilm formation. Biofilm strength was inversely proportional to HC dilution (*p*-value < 0.0001). Nevertheless, HC and colostrum-derived probiotics improve oral health by inhibiting the growth of caries-inducing *S. mutans* with lower inhibition to *L. rhamnosus* probiotics. The CFS of isolated probiotics reduced the biofilm formation via the cariogenic *S. mutans*. These results are not only promising for caries eradication, but they also highlight the importance of breastfeeding infants from their first hours to shape healthy oral microbiota, protecting them from various diseases including dental caries.

## 1. Introduction

Despite being multifactorial, involving injudicious eating habits and poor oral health, dental caries remains a first-rate microbiological disease. Bacterial biofilm is the initial caries precursor and *Streptococcus mutans* (*S. mutans*) is the major contributor [1,2]. Inhibition of *S. mutans* and the associated biofilm, therefore, could hinder the caries process. *S. mutans* is both acidogenic and aciduric. Moreover, *S. mutans* synthesizes glucan and fructan from sucrose by the action of glucosyltransferase and fructosyltransferase, respectively [3]. These exopolysaccharides increase bacterial retention on tooth surfaces with subsequent demineralization. Various strategies have been adopted for inhibiting *S. mutans*; the use of probiotics is one of the most recent ones [4,5].

Probiotics are microorganisms that can hinder other pathogens, enhancing the quality of the microbiota and preserving a stable eubiosis; thus, they can prevent or even cure diseases [6,7]. Probiotics are suggested to exert their action via competition with the pathogen for nutrition and binding sites, and/or enhancing arginine deiminase system activity. The latter forms an alkaline ecology unfavorable for aciduric cariogenic microorganisms [8]. Probiotics could be obtained from several natural sources such as yogurt, kefir, pickles, and some types of cheese, and they are present in the mouth and colostrum of Mammalia [9]. 

Colostrum is the first milk type produced by mammals immediately postpartum [10]. It includes an abundance of immune proteins such as immunoglobulins, lactoferrin, and numerous cytokines that resist digestion, prolonging their biological activity. Colostrum also contains oligosaccharides that may serve as a selective food source (prebiotic) and favor the growth of various beneficial bacteria [11]. Additionally, it was proven that human colostrum has useful bacteria that can serve as probiotics as well [12]. 

It is well known that human milk has cariogenic activity [13]. As *S. mutans* were also detected in the saliva of some predentate children [11], studies are needed to evaluate the impact of human colostrum (HC) on dental health. Therefore, this study was carried out to evaluate the effect of HC and their derived probiotics’ cell-free supernatants (CFSs) on oral health by assessing their effect on the growth of *S. mutans* and their biofilm. Moreover, the effect of whole HC on *L. rhamnosus*, a probiotic oral bacterium, is carried out. The colostrum and their derived probiotics, if proven effective, could be used in dental health prophylaxis [5].

## 2. Materials and Methods

### 2.1. Ethical Approval

The study protocol was accepted by the Committee of Research Ethics of the Faculty of Dental Medicine for Girls, Al-Azhar University, Cairo, Egypt (P-PD-22-13). This study coincides with the Declaration of Helsinki guidelines [14].

### 2.2. Sample Size Calculation

The calculation of the sample size was guided by the Kim et al. study [15]. According to this study, the minimally accepted sample size was 29. When the response within each subject group was normally distributed with a standard deviation of 1.3, the estimated mean difference was 1 when the power was 80% and the type I error probability was 0.05. The sample size was increased to 36 to compensate for the 20% dropout. The sample size was calculated using P.S.power 3.1.6.

### 2.3. Collection of HC Samples

Following written consent, about 30 mL of HC was collected from each of the 36 healthy mothers 1–3 days postpartum. It was reported that all contributors had received antibiotics after delivery. The samples were cooled till transferred to the lab within 4 h, centrifuged (3000 rpm for 10 min at 4 °C), filter-sterilized (0.45 µm) (Millipore, Bedford, MA, USA), and kept at −20 °C until used [16]. 

### 2.4. Bacterial Strains and Growth Conditions

*S. mutans* strain ATTC 25175 was isolated from dentin caries, cultured at 37 °C on brain-heart infusion (BHI) medium (Oxoid Ltd., Hampshire, UK), and then incubated for 48 h in 5% CO_2_ at 37 °C. DeMan, Rogosa, Sharpe (MRS) agar and broth (Oxoid Ltd.), however, were used for the isolation of probiotic bacteria from HC. Cultures were incubated for 24 h in 5% CO_2_ at 37 °C [3,17]. 

### 2.5. Effect of the Whole HC on the Oral Bacteria S. mutans, and L. rhamnosus

The effect of the whole HC was examined using the modified well diffusion method [18] to learn the effect on the growth of the oral bacteria *S. mutans* (*ATCC* 25175), and *L. rhamnosus* (ATCC 7469) (probiotic bacteria that inhabit the mouth and protect against tooth decay via *S. mutans* [19]). A total of 20 mL of *S. mutans* or *L. rhamnosus* suspension (1.5 × 10^8^ CFU/mL) was added to 200 mL of melted MRS agar and kept at 45 °C. The mix was poured into plates and left to solidify. A total of 150 µL of HC was added to the wells, while other wells contained 2% chlorhexidine acetate, the standard antibacterial, as a positive control (Madam Health Pharmaceutical Co., Ltd., Shanghai, China). After incubation (24 h at 37 °C), the growth inhibition diameters were determined [4].

### 2.6. Effect of the Whole HC on S. mutans Biofilm Formation

The microtiter plate assay (MTP) was performed as dictated by Allison et al. (2015) [16]. Various dilutions of HC (1:3, 1:9, 1:27, 1:81, 1:243, 1:729, and 1:2187) were investigated. HC samples were mixed with BHI (190 µL total volume) and 10 µL of an overnight culture of *S. mutans* (10^6^ CFU/mL) in seven rows (one row/each dilution) out of eight of the 96-well-flat bottom sterile polystyrene microtiter plates for 24 h. BHI with and without *S. mutans* were used as positive and negative controls, respectively, in the remaining row of wells. After incubation, each well was triple-washed (300 µL of phosphate buffer saline), heat-fixed (60 °C for 1 h), crystal violet-stained (150 µL for 15 min), and the stain was extracted by absolute ethanol (150 µL) (Merck, Darmstadt, Germany). Finally, stained adherent biofilms were graded, as shown in Table 1. According to Pui et al. (2017) and Salem et al. (2022), depending on their optical densities, an OD of 630 nm was recorded by a Stat Fax^®^ 2100 microtiter reader (Awareness Technologies Inc., Westport, CT, USA). The test was repeated three times and the results were averaged [20,21]. 

### 2.7. HC Probiotics Isolation and Identification

Of each HC sample, 10 µL was cultured in sterile 5 mL MRS broth (72 h, 5% CO_2_ at 37 °C), then sub-cultured twice on MRS agar plates to obtain pure colonies in case of the presence of mixed colonies. Samples were first cultured on broth to maximize the yield as initial culturing of HC on MRSA agar plates did not show any bacterial growth. The bacterial growth was indicated by showing bacterial colonies. The resultant colonies were initially identified by their morphology on MRS agar plates, Gram staining, and catalase reaction. Catalase-negative isolates were species identified using API 50 CH carbohydrate fermentation strips [22] (bioMerieux, Craponne, France) and 16S ribosomal ribonucleic acid 16S rRNA [23]. Following the isolates’ genomic DNA extraction using NucliSENS easyMAG (bioMerieux), 16S rRNA genes were PCR amplified. The amplicons were sequenced in Cosmo Gentech, Seoul, Republic of Korea, using an Applied Biosystems 3730x1 DNA Analyzer (Thermo Fisher, Seoul, Republic of Korea). Isolates with the highest Basic Local Alignment Search Tool (BLAST) score were identified against the GenBank DNA database (www.ncbi.nlm.nih.gov/Genbank, accessed on 10 November 2022) [24,25]. 

### 2.8. Preparation of Cell-Free Supernatant (CFS) of Isolated Probiotics

For CFS preparation, each probiotic isolate was inoculated into 30 mL of MRS broth at (10^8^ CFU/mL) concentration, followed by incubation (24 h in 5% CO_2_ at 37 °C), centrifugation (4500 rpm for 10 min at 4 °C), and filtration (0.45 µm syringe filter) (Millipore) [26]. 

### 2.9. Anti-S. mutans Effect of CFS of Isolated Probiotics

Using the modified well diffusion method [18], 20 mL of *S. mutans* suspension (1.5 × 10^8^ CFU/mL) was added to 200 mL of melted MRS agar and kept at 45 °C. The mix was poured into plates and left to solidify. The CFS (150 µL) of isolated probiotics were added to the wells, while other wells contained 2% chlorhexidine acetate, the standard antibacterial, as a positive control (Madam Health Pharmaceutical Co., Ltd., Shanghai, China). After incubation (24 h at 37 °C), the *S. mutans* growth inhibition diameters were determined [4]. 

### 2.10. Anti-S. mutans Biofilm Effect of CFS of Isolated Probiotics (MTP Assay)

The previously mentioned MTP assay was used for assessing the effect of the CFS of the isolated probiotics on *S. mutans* biofilm formation. The CFS of each isolated bacteria were used in a dilution that did not inhibit the growth of *S. mutans* [27]. The ODs of stained retained biofilms were triple assessed [26] using a microtiter plate reader, at an OD of 570 nm. The percentages of biofilm inhibition were calculated according to the following equation [28]:

% inhibition = (1 − OD sample/OD positive control) × 100.

### 2.11. Statistical Analysis

Statistical analysis was performed with SPSS 20^®^, Graph Pad Prism^®^, and Microsoft Excel 2016. All quantitative data were explored for normality using a Shapiro–Wilk normality test and presented as means and standard deviation (SD) values. Qualitative data, however, were presented as frequencies and percentages.

## 3. Results

### 3.1. The Cariogenic Activity of HC

#### Effect of the Whole HC on *S. mutans* Biofilm Formation

**Figure 1:**
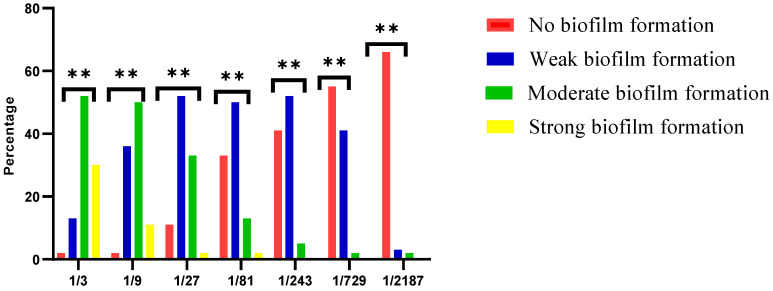
Effect of different dilutions of the whole HC on *S. mutans* biofilm formation. **: Significance.

Whole HC samples were associated with biofilm formation; the biofilm strength was inversely proportional to HC dilution. The frequencies and percentages of *S. mutans* biofilm formation of different HC dilutions are shown in Figure 1. A Chi-square test revealed a significant difference among different HC dilutions (*p* ≤ 0.05). 

### 3.2. The Anti-Caries Potential of HC

#### 3.2.1. Effect of the Whole HC on the Oral Bacteria *S. mutans* and *L. rhamnosus*

**Figure 2:**
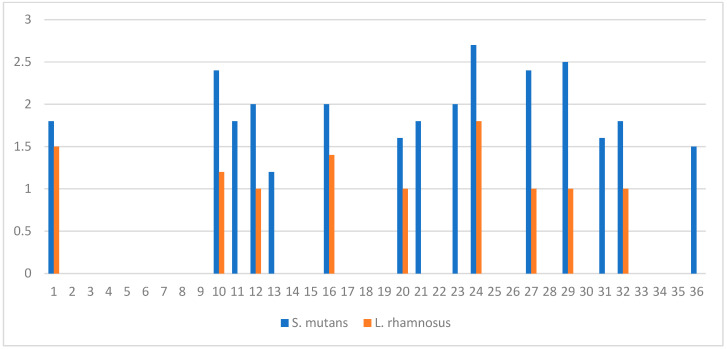
Inhibition zone diameters (cm) of whole colostrum against *S. mutans* (blue pars), and *L. rhamnosus* (orange bars) for all collected colostrum (n = 36). Well diameter = 0.8 cm.

The well-diffusion test showed that HC inhibits the growth of the cariogenic bacteria, *S. mutans*, with inhibition zone diameters ranging from 12 to 27 mm, while the inhibition zone diameters for the probiotic bacteria, *L. rhamnosus*, ranged from 10 to 18 mm (Figure 2). 

#### 3.2.2. Identification of Probiotic Bacteria Isolated in MRS from HC 

From nine colostrum samples, ten bacteria were isolated in MRS agar and phenotypically identified: *Lactobacillus rhamnosus* (n = 1), *Pediococcus acidilactici* (n = 2), *Pediococcus pentosaceus* (n = 1), *Leuconostoc lactis* (n = 1), *Leuconostoc mesenteroides* (n = 1), *Lactococcus lactis* ssp *lactis* 1 (n = 1), *Staphylococcus lugdunensis* (n = 1), *Pseudomonas helleri* (n = 1), and *Staphylococcus epidermidis* (n = 1). Genotypically identified species were recorded in the GenBank DNA database, along with their accession numbers, and are listed in Table 2. 

#### 3.2.3. Anti-*S. mutans* Effect of CFS of Isolated Probiotics

Most isolated (7/10) probiotics have successfully inhibited the growth of *S. mutans* with variable degrees. The difference among different isolated probiotics regarding *S. mutans* inhibition was statistically insignificant (*p* = 0.51) according to one-way ANOVA (*p* > 0.05). The effect of CFS of isolated probiotics on the growth of *S. mutans*, was presented as mean and standard deviation values of inhibition zone diameters (in mm) as shown in Table 3.

#### 3.2.4. Anti-*S. mutans* Biofilm Effect of CFS of Isolated Probiotics

The CFS of isolated probiotics showed anti-*S. mutans* biofilm efficacy that varied among different probiotic species. The difference among them, however, was statistically insignificant (*p* = 0.14), as evidenced by one-way ANOVA (*p* > 0.05). The mean and standard deviation values of % inhibition of *S. mutans* biofilm formation by different isolated bacteria CFS are presented in Table 4 [15,28].

## 4. Discussion

Dental caries is still a widespread public health disease, even with progress in treatment strategies. Nowadays, most trends are directed toward prevention [29]. Being the outcome of host/microbiome imbalance [30], caries could be prevented as early as possible via modifying oral microbiomes and controlling caries-implicated strains such as *S. mutans* [31]. There is a growing interest in using probiotics, especially naturally driven species, for this purpose [30]. 

Breast milk was evidenced to include a diverse bacterial community that could formulate the infant’s microbiome [23]. The inclusion of more than 200 bacterial species makes breast milk a good source of natural probiotics [28]. HC was selected as a source of probiotics as the first administered nutrient capable of altering the oral ecology [32]. This study investigated the effect of different dilutions of HC on the formation of biofilm by cariogenic *S. mutans*. Results showed that almost all HC samples increased *S. mutans* biofilm formation. Biofilm strength was inversely proportional to the degree of HC dilution. This could be attributed to the fact that human milk, as a whole, contains both cariogenic (e.g., casein) and anti-cariogenic constituents (e.g., IgA and probiotics), as evidenced in the Allison et al. study [16]. Such results are further supported by the occurrence of nursing caries, a disease in which dental caries cause severe damage to the anterior teeth of the suckling baby as a result of prolonged breastfeeding, esp. during sleeping at night, in the absence of the mother’s awareness of oral hygiene [33].

The anticaries potential of HC was also demonstrated in this study by revealing that whole colostrum inhibited the growth of cariogenic *S. mutans* with inhibition zone diameters ranging from 12 to 27 mm. This inhibitory activity could be attributed to its constituents of probiotic bacteria and secretory immunoglobulin (IgA) [11,12]. Surprisingly, this bacterial inhibitory activity was lower for the beneficial oral probiotic *L. rhamnosus* as their zone of inhibition ranged from 10 to 18 mm. Some HC specimens that inhibited *S. mutans* did not affect the growth of *L. rhamnosus*, which is an added advantage indicating that HC had preserved this probiotic bacterium, an effect that has a positive impact on oral health and oral microbiome. 

It is noted that only 25% (9/36) of HC showed antibacterial activity. Only ten beneficial bacteria including *Lactobacillus rhamnosus* (n = 1), *Pediococcus acidilactici* (n = 2), *Pediococcus pentosaceus* (n = 1), *Leuconostoc lactis* (n = 1), *Leuconostoc mesenteroides* (n = 1), *Lactococcus lactis* ssp *lactis 1* (n = 1), *Staphylococcus lugdunensis* (n = 1), *Pseudomonas helleri* (n = 1), and *Staphylococcus epidermidis* (n = 1) were isolated from the HC of Egyptian mothers. Colostrum is less diverse than mature milk [28]. This could explain obtaining only 10 isolates belonging to six genera from HC samples: *Pediococcus* (30%, 3/10), *Leuconostoc* (20%, 2/10), *Lactococcus* (10%, 1/10), *Lactobacillus* (10%, 1/10), *Staphylococcus* (20%, 2/10), and *Pseudomonas* (10%, 1/10). This complies with the results of other studies [28,34]. Although *Lactobacillus* and *Bifidobacterium* are usual probiotics of breast milk, they formed only 0.4% and 1.7% of isolated probiotics. This was also shown by Chen et al. (2020), who have even recorded their absence in some milk samples [6]. Breast milk microbiome could be individually varied according to genetics, diet, lifestyle, lactation period, and way of delivery [35]. This reported low count of isolated bacteria is in agreement with another study that mentioned that due to low microbial DNA amounts or sterility, 35% of breast milk from healthy postpartum women could not be isolated or even sequenced. In addition, systemic antibiotic administration to almost all Egyptian mothers after delivery might have reduced microbial count and diversity in HC samples [23]. The sterility of most HC isolated from Egyptian mothers is attributed to inappropriate use of antibiotics after delivery, as the misuse of antibiotics leads to the destruction of microbiota in addition to the emergence of antibiotic resistance [36,37]. These results showed the importance of antibiotic restriction, especially postpartum, so that the newborn benefits from varieties of the mother’s microbiomes that would positively affect their dental as well as general health. Several studies reported the effect of antibiotics on the microbiome [38]. Specific studies are needed to determine the effect of antibiotics administration on the composition of human colostrum and human breastmilk and their relation to oral health and the emergence of resistant genes.

In the current study, diluted CFS of most isolated probiotics has inhibited *S. mutans* and its biofilm formation by different grades. *L. rhamnosus*, for example, caused 34.58 ± 1.91% biofilm inhibition following the Kim et al. study, where CFS of *L. rhamnosus* and *L. brevis* had inhibited the biofilm formation by 41.45% and 35.28%, respectively [39]. Most *Lactobacillus* strains are known to exhibit anti-*S. mutans* activity by their bioactive agents [40]. However, in agreement with other studies, the current study revealed that in addition to *Lactobacillus*, *S. mutans* biofilm was inhibited by other probiotic species such as *Pediococcus pentosaceus*, *Pediococcus acidilactici*, *Leuconostoc mesenteroides*, *Leuconostoc lactis*, and *Lactococcus lactis* ssp *lactis 1* [34,39,41]. 

On the other hand, isolated *Staphylococcus epidermidis, Staphylococcus lugdunensis*, and *Pseudomonas helleri* exhibited limited efficacy. Nevertheless, they are still capable of fighting *S. mutans*, antagonizing it, and preventing colonization [42,43,44]. Moreover, they could be used for the isolation of new antibiotics with proven bactericidal effects [45,46]. The abovementioned effects support our findings in which *S. mutans* biofilm formation was inhibited by the CFS of *Staphylococcus epidermidis*, *Staphylococcus lugdunensis*, and *Pseudomonas helleri* by 16.67, 22.92, and 22.92% respectively, confirming that HC and human milk are potential sources for obtaining solution for different health problems. Although the fact that human milk and human colostrum have some cariogenic activity due to their content of sucrose [47], they are rich sources of probiotic bacteria that maintain oral ecology and oral microbiome. In a previous study that examined the effect of probiotic bacteria on *S. mutans* and *C. albicans* using a thorough multispecies biofilm model that simulated high caries risk clinical situations, they found that probiotics inhibited biofilm formation and growth of *S. mutans* and *C. albicans* and suppressed the components of exopolysaccharides. EPS production, carbohydrate metabolism, glycan biosynthesis, and metabolism-related genes in *S. mutans* and *C. albicans* were dramatically downregulated. More importantly, there was also a considerable downregulation of genes associated with *C. albicans* resistance to antifungal drugs (ERG4), fungal cell wall chitin remodeling (CHT2), and resistance to oxidative stress (CAT1). The antimicrobial peptide plantaricin is produced by the Lactobacillus genes plnD, plnG, and plnN, which were considerably elevated [48]. 

The results of the present study revealed the in vitro cariogenic activity of HC by enhancing biofilm formation of *S. mutans*. At the same time, whole HC provides anticaries potential and is a source of probiotic and beneficial bacteria that fight *S. mutans* and its biofilm formation. An interesting finding is that the whole HC did not inhibit *S. mutans* biofilm formation, while isolated probiotics showed different degrees of cariogenic *S. mutans* inhibition. HC contains both cariogenic components (e.g., casein) and anti-cariogenic ones (such as probiotics and immunoglobulins). The cariogenic potential of milk and its substitutes directly depends on the way they are used. Caries is a multi-factorial disease requiring the presence of fermentable carbohydrates, cariogenic bacteria, and caries-susceptible surface (i.e., tooth surface). In the case of newborns, there are no teeth; accordingly, they can benefit from the probiotics in the colostrum in formulating healthy oral microbiota with no caries susceptibility till teeth eruption. The already-shaped healthy oral microbiota would later decrease caries risk, even after teeth eruption. It is noteworthy that in our in vitro study, the strength of the biofilm formed in the whole HC test was inversely proportional to HC dilution, probably due to the dilution of both cariogenic and anti-cariogenic components. It is therefore logical that, when the anti-cariogenic probiotics were isolated, the inhibition of *S. mutans* was more pronounced [1,2,49].

Probiotic supplements and a prebiotic diet are encouraged for children who are deprived of natural breastfeeding to restore their oral microbiome and ensure good oral and dental health [50,51]. To reverse the undesired changes in the microbiota’s composition and function brought on by antibiotic treatments or caesarean delivery, a probiotic mixture in addition to at least partial breastfeeding is highly recommended [52]. More studies are needed to report the effect of antibiotic misuse in Egypt on several aspects of health, including oral health. This study shed a light on the benefits of the probiotics on oral health in addition to the previously known effects on intestinal health or prevention of toxicity [53,54].

The limitations of this study involved the inability to use the metagenomic analysis that might reveal several microorganisms other than bacteria that help in restoring oral ecology. More studies targeting the details of HC’s whole components and in vivo studies are needed to confirm the impact of HC on dental health. Moreover, retrospective studies are encouraged to evaluate the oral health of individuals who had received human milk in their early life. Analysis of human colostrum and cell-free supernatants of isolated probiotics will add a significant discovery to understanding the power of human milk.

## 5. Conclusions

The present study’s findings demonstrate the in vitro cariogenic activity of HC by encouraging *S. mutans* to produce biofilms. On the other hand, CFS of isolated HC probiotics was proven to have good anti-*S. mutans* and antibiofilm effects; opening the way for early oral microbiome control and pursuing caries uprooting while emphasizing the importance of breastfeeding from the first hours of an infant’s life. Other in vivo research with greater sample sizes assessing *S. mutans* in the saliva of newborns before and after colostrum administration, microbiome alteration after the transition to mature breast milk, and comparing colostrum to mature breast milk after a normal delivery is highly advocated. 

## Figures and Tables

**Table 1:** Grading of biofilm formation.

Biofilm Formation	Characterization
Grade 0	Absent (OD ≤ ODc).
Grade 1	Weak (ODc < OD ≤ 2ODc).
Grade 2	Moderate (2ODc < OD ≤ 4ODc).
Grade 3	Strong (4ODc < OD).

OD = average optical density of the tested samples, ODc = average OD of negative control + 3SD of the negative control.

**Table 2:** Accession numbers of genotypically identified isolated probiotics.

Species	Accession Number
*Pediococcus acidilactici*	OP647353.1
*Pediococcus acidilactici*	OP647354.1
*Pediococcus pentosaceus*	OP647355.1
*Staphylococcus lugdunensis*	OP647356.1
*Pseudomonas helleri*	OP647357.1

**Table 3:** Effect of CFS of isolated probiotics on the growth of *S. mutans*.

Isolated Probiotic Species	Mean of Inhibition Zone Diameters in cm	Standard Deviation (SD)	N.B.
*Leuconostoc lactis*	1.77	0.15	Isolated from sample No. 2
*Leuconostoc mesenteroides*	1.87	0.12	Isolated from sample No. 10
*Lactobacillus rhamnosus*	1.87	0.21	Isolated from sample No. 16
*Pediococcus acidilactici*	1.73	0.12	Isolated from sample No. 23
*Pediococcus acidilactici*	1.77	0.15	Isolated from sample No. 24
*Lactococcus lactis* ssp *lactis 1*	1.77	0.15	Isolated from sample No. 24
*Pediococcus pentosaceus*	1.97	0.15	Isolated from sample No. 27
*Staphylococcus lugdunensis*	-	-	Isolated from sample No. 29
*Pseudomonas helleri*	-	-	Isolated from sample No. 31
*Staphylococcus epidermidis*	-	-	Isolated from sample No. 32

CFS: cell-free supernatant. (-): no inhibition zone.

**Table 4:** Effect of CFS of isolated bacteria on % inhibition of biofilm formation by *S. mutans*.

Species	Mean	Standard Deviation (SD)
*Leuconostoc lactis*	31.25	3.31
*Leuconostoc mesenteroides*	28.75	9.92
*Lactobacillus rhamnosus*	34.58	1.91
*Pediococcus acidilactici*	35.83	4.39
*Pediococcus acidilactici*	37.92	0.72
*Lactococcus lactis* ssp *lactis 1*	32.08	6.88
*Pediococcus pentosaceus*	35.83	5.64
*Staphylococcus lugdunensis*	22.92	16.12
*Pseudomonas helleri*	22.92	16.12
*Staphylococcus epidermidis*	16.67	10.41

CFS: cell-free supernatant. CFS from each isolated bacteria are used in a dilution that does not inhibit the growth of *S. mutans*.

## Data Availability

All data generated during this study are included in the published article.
